# Characterization and Functional Analysis of Four Glutathione *S*-Transferases from the Migratory Locust, *Locusta migratoria*

**DOI:** 10.1371/journal.pone.0058410

**Published:** 2013-03-07

**Authors:** Guohua Qin, Miao Jia, Ting Liu, Xueyao Zhang, Yaping Guo, Kun Yan Zhu, Enbo Ma, Jianzhen Zhang

**Affiliations:** 1 Research Institute of Applied Biology, Shanxi University, Taiyuan, Shanxi Province, China; 2 The College of Environmental Science and Resources, Shanxi University, Taiyuan, Shanxi Province, China; 3 Department of Entomology, Kansas State University, Manhattan, Kansas, United States of America; University of Tennessee, United States of America

## Abstract

Glutathione *S*-transferases (GSTs) play an important role in detoxification of xenobiotics in both prokaryotic and eukaryotic cells. In this study, four GSTs (LmGSTd1, LmGSTs5, LmGSTt1, and LmGSTu1) representing different classes were identified from the migratory locust, *Locusta migratoria*. These four proteins were heterologously expressed in *Escherichia coli* as soluble fusion proteins, purified by Ni^2+^-nitrilotriacetic acid agarose column and biochemically characterized. LmGSTd1, LmGSTs5, and LmGSTu1 showed high activities with 1-chloro-2, 4-dinitrobenzene (CDNB), detectable activity with p-nitro-benzyl chloride (p-NBC) and 1, 2-dichloro-4-nitrobenzene (DCNB), whereas LmGSTt1 showed high activity with p-NBC and detectable activity with CDNB. The optimal pH of the locust GSTs ranged between 7.0 to 9.0. Ethacrynic acid and reactive blue effectively inhibited all four GSTs. LmGSTs5 was most sensitive to heavy metals (Cu^2+^ and Cd^2+^). The maximum expression of the four GSTs was observed in Malpighian tubules and fat bodies as evaluated by western blot. The nymph mortalities after carbaryl treatment increased by 28 and 12% after LmGSTs5 and LmGSTu1 were silenced, respectively. The nymph mortalities after malathion and chlorpyrifos treatments increased by 26 and 18% after LmGSTs5 and LmGSTu1 were silenced, respectively. These results suggest that sigma GSTs in *L. migratoria* play a significant role in carbaryl detoxification, whereas some of other GSTs may also involve in the detoxification of carbaryl and chlorpyrifos.

## Introduction

Glutathione *S*-transferases (GSTs) are multifunctional enzymes involved in detoxification of xenobiotics in both prokaryotic and eukaryotic cells. In general, GSTs act by conjugating the thiol group from glutathione (GSH; γ-glutamyl-cysteinyl-glycine) to compounds that possess an electrophilic center. By this mechanism, they can eliminate substrates from a cell by rendering them more water soluble and targeting those to specific GSH multidrug transporters. In insects, GSTs can be separated into two major groups: microsomal and cytosolic GSTs. The membrane-bound microsomal GSTs are structurally and evolutionarily distinct from the cytosolic GSTs [1]. The cytosolic GSTs are further classified into six major classes along with several unclassified genes [1]. Among them, sigma, omega, zeta, and theta have representatives across Metazoa whereas delta and epsilon are specific to Insecta and Holometabola, respectively [2]. In spite of low sequence homology among GST classes they have fairly similar tertiary structures, topography of active site and G-sites, and are inducible by certain insecticides and other chemicals [1], [3]. Most GSTs are cytosolic and, present in both homo and heterodimeric forms with subunit masses of 23- to 28-kDa [4]. Each subunit contains two domains and one active site; and within the active site there are two binding sites, one for GSH and other for hydrophobic substrate [5], [6]. GSTs act on different substrates and can protect insects against various plant allelochemicals and chemical insecticides. However, not all the insect GSTs are involved in detoxification [7]. They carry out a wide range of functions in cells, such as the removal of reactive oxygen species and regeneration of *S*-thiolated proteins (both of which are consequences of oxidative stress), catalysis of conjugations with endogenous ligands, and catalysis of reactions in metabolic pathways not associated with detoxification [8].

Although many GST cDNAs have been sequenced from different insect species, little is known about functional specificities of GSTs in different classes. In mosquitoes, GSTs are characterized to play a role in metabolism of DDT (dichloro-diphenyl-trichloroethane) [9]. And GSTs show possible protective roles against oxidative damage caused by the pyrethroids in *Nilaparvata lugens* [10]. The epsilon class GSTs from *Spodoptera litura* and *Anopheles gambaie* might be capable of detoxifying DDT and/or deltamethrin [11], [12].

The migratory locust, *Locusta migratoria*, is the most widespread locust species. It occurs throughout Africa, Asia, Australia and New Zealand. Frequent applications of insecticides have inevitably resulted in development of resistance in some natural populations of the locust [13]–[16]. However, little is known about the role of GSTs of *L. migratoria.* By searching the *L. migratoria* EST databases, we have identified 10 putative cytosolic GSTs, among which nine fall into three classes (delta, sigma, and theta), and the remaining one does not fit any of known GST classes and is tentatively designated as unclassified [17]. We previously reported that one of the sigma class GSTs from *L. migratoria* might be capable of detoxifying carbaryl [18]. In this study, four GSTs representing four different classes including one delta GST (LmGSTd1), one sigma GST (LmGSTs5), one theta GST (LmGSTt1), and one unclassified GST (LmGSTu1), were heterologously expressed as recombinant enzymes in *Escherichia coli* cells. We further characterized various biochemical properties of these recombinant proteins and assessed their detoxification functions against five insecticides by RNA interference (RNAi). Our results are expected to help researchers better understand biochemical properties and detoxification functions of locust GSTs.

## Materials and Methods

### Insect

*L. migratoria* were purchased from the Insect Protein Co., Ltd. of Cangzhou City in China and reared in the laboratory with wheat sprouts in 22×22×22 plastic cages at 28°C under 14∶10 h light : dark photoperiod.

### Construction and Production of the Recombinant Plasmids

The full cDNA sequences of *L. migratoria* GSTs were obtained in our previous study [16]. The cDNA sequences were analyzed by ExPASy (http://www.expasy.ch/) to deduce the amino acid sequence, predict protein molecular mass and *pI*. Open reading frames (ORF) of the four GST cDNAs were amplified by PCR with corresponding primers (Table S1). PCR was conducted for one cycle at 95°C for 3 min; 35 cycles, each at 94°C for 30s, 55°C for 30s, and 72°C for 1 min; and followed by one cycle at 72°C for 7 min. The amplified products were inserted into the pGEM-T Easy vector (Promega, Madison, WI, USA), and the plasmids were digested with restriction enzymes as shown in Table S1. Resulting digests were subcloned into an expression vector, pET-28a (Novagen, Madison, WI, USA). The recombinant plasmids were named pET-28a-LmGSTd1, pET-28a-LmGSTs5, pET-28a-LmGSTt1, and pET-28a-LmGSTu1. All constructs were confirmed by DNA sequencing. The recombinant plasmids harboring LmGSTs were used to transform *E. coli* BL21 (DE3) or JM109 (Invitrogen), which were grown at 37°C on Luria-Bertani (LB) media containing 100 µg/mL ampicillin. After the cell density reached 0.6–0.8 at OD_600_, isopropyl 1-thio-β-D-galactoside (IPTG) was added to a final concentration of 1 mM to induce the production of recombinant proteins.

After further incubation for 4 h, cells from a 1-L culture were harvested by centrifugation, and the resulting pellet was resuspended in 90 ml 50 mM PBS buffer (pH 8.0) containing 0.5 M NaCl, 0.1% Triton X-100, and 0.05% Tween 20. The cell suspension was sonicated and centrifuged at 15,000×g at 4°C for 30 min. The supernatant (cleared lysate) was transferred to 50% slurry Ni^2+^- nitrilotriacetic acid (NTA) agarose beads (Qiagen, Valencia, CA, USA) that were pre-equilibrated with above PBS. The Ni-NTA resin was sequentially washed using 20 ml PBS buffer with a linear gradient of imidazole from 5 to 250 mM. The recombinant *L. migratoria* GSTs was eluted with PBS containing 250 mM imidazole and dialyzed against TGE buffer (50 mM Tris, 0.5 mM EDTA, 50 mM NaCl, 5% glycerine, 1% glycine, pH 8.0). The purity of LmGSTs was evaluated by 12% sodium dodecyl sulfate-polyacrylamide gel electrophoresis (SDS-PAGE).

### GST Activity Assays

The enzyme activity of LmGST recombinant proteins were assayed as described by Qin et al. [18]. Ten microliters (10 µg) of protein was used in a total volume of 200 µL of a reaction mixture. The two substrates for GST, 1-chloro-2, 4-dinitrobenzene (CDNB) and reduced glutathione, were added to the reaction wells. The change in absorbance of CDNB conjugate for the first minute was measured at 340 nm and 28°C, with 10-s intervals using Multiple Mode Microplate Reader SpectraMax M5 (ε340 = 9600 M^−1^ cm^−1^) (Molecular Devices Corporation, Sunnyvale, CA, USA). Controls were performed in parallel in order to correct for nonenzymatic conjugation of GSH to the substrates. Protein concentration was determined according to the method of Bradford using bovine serum albumin as a standard (0–4 mg/ml) [19]. Enzyme activity is presented as nmol of CDNB conjugated per min per mg protein. The apparent K_m_ and V_max_ were determined for each of four purified GSTs using non-linear regression of hyperbolic plots (V versus S). Each data point represents the average of 3 measurements. When other substrates were used in the assay for GST under the same conditions described above, changes in absorbance per min were converted into nanomoles of the substrate conjugated/min/mg protein using the molar extinction coefficient: ε345 = 8500 M^−1^ cm^−1^ for 1,2-dichloro-4-nitrobenzene (DCNB), ε310 = 1900 M^−1^ cm^−1^ for p-nitro-benzyl chloride (p-NBC), and ε270 = 5000 M^−1^ cm^−1^ for ethacrynic acid (ECA).

For the assay of *in vitro* inhibition of GST, the enzyme activities were measured at 28°C in a total volume of 200 µL of 0.1 M phosphate buffer (pH 7.5) containing 2 mM CDNB and 5 mM GSH in the presence or absence of 10 µL of appropriately diluted inhibitors, or heavy metal solutions.

### Western Blot Analysis

Total protein was extracted from each of 9 different tissue samples, including foregut, midgut, gastric caecum, hindgut, Malpighian tubules, fat bodies, muscles, spermaries and ovaries, dissected from fifth-instar nymphs in ice-cold lysis buffer (1% Nonidet P40, 1 mM EDTA, 125 mM sodium fluoride, 0.5 mM sodium vanadate, 2.5 µg/mL of aprotinin, 5 µg/mL of pepstatin, 50 µg/mL of leupeptin, 25 µM PMSF, and 25 µg/mL of Trypsin inhibitor). Protein concentration was determined according to the method of Bradford using bovine serum albumin as a standard [19].The lysates were centrifuged at 13,000 rpm for 15 min and supernatants were collected. Purified LmGST proteins were emulsified with Freund's complete adjuvant and injected subcutaneously into two 6-month old male rabbits. Booster injections of LmGSTs, emulsified with Freund's incomplete adjuvant were also administered. Serum was collected after the second booster and IgG was purified using Protein-A-affinity chromatography (Bio-Rad). The specificity of the primary antibody were detected (Figure S1). SDS-PAGE was performed using 150 µg protein samples and precast 12% resolving and 4% stacking Tris-HCl gels (Bio-Rad). Separated proteins were then transferred to a *nitrocellulose membrane* (Millipore, Billerica, MA). After blocking (blocking solution: 5% non-fat milk dissolved in PBS +0.1% Tween 20, pH 7.4) proteins were incubated overnight at 4°C with anti-GST antibodies, at a concentration of 1∶200 (for GSTd1), 1∶500 (for GSTt1), or 1∶5000 (for GSTs5 and GSTu1). Exposure to fluorescently labeled secondary antibody (1∶3000) [IRDye 680CW goat anti-rabbit IgG (H+L), LI-COR] was followed by scanning and detecting with LI-COR Odyssey Infrared Fluorescent System.

### Synthesis of dsRNA and Performance of RNAi

Double-stranded RNA (dsRNA) was synthesized and RNAi was performed as described in an established protocol [18]. Briefly, C-terminal alpha helical domain fragment of each *L. migratoria GST* was obtained by PCR from the full-length cDNA clone using sequence-specific primers (Table S1). dsRNA was synthesized using T7 RiboMAX Express RNAi System (Promega) according to manufacturer’s instructions. Then, 2 µL of dsRNA (1.5 µg µL^−1^) from target genes or green fluorescent protein (GFP) control was injected into the abdomen between the second and third abdominal segments of each second-instar nymph (3 days old) by using a microsyringe. The efficiency of RNAi was examined by quantitative reverse transcription PCR (qRT-PCR) using specific primers (Table S1). For each locust GSTs RNAi assay, 250–300 nymphs were injected with dsRNA of GFP or GSTs. The nymphs from either control or treatment group were reassigned into 5 different insecticides groups, and each was exposed to each of five different insecticides including DDT, chlorpyrifos, carbaryl, deltamethrin, and malathion at 24 h after injection. About 50–60 nymphs from the control or locust GSTs dsRNA- injected group were randomly divided into three subgroups, each with 15–20 insects as a biological sample for each insecticides bioassay. And a droplet of 2 µL of acetone containing DDT (220 ng/µL), chlorpyrifos (6.5 ng/µL), carbaryl (17 ng/µL), deltamethrin (0.5 ng/µL), or malathion (85 ng/µL), was topically applied onto the abdomen between the second and third sterna of each nymph. Mortality was recorded at 24 h after topical applications. Nymphs were considered dead if they were not able to move in a coordinated way when touched with a brush.

## Results

### Sequence Analysis of *L. migratoria* GSTs

The detailed information of complete cDNA, the predicted molecular mass, and their estimated p*I* of four *L. migratoria* GSTs are summarized in Table 1. The complete cDNA of LmGSTs are from 680 to 1100 bp. The open reading frames (ORF) are from 609 to 696 bp with a coding capacity of 202–231 amino acid residues. The predicted molecular masses of these deduced proteins are from 23.1 to 26.6 kDa. The estimated *pI* values range from 5.57 to 7.76.

**Table 1:** Summary of molecular properties of four *L. migratoria* GSTs.

GenBank accession number	Gene name	Length of cDNA (bp)	ORF(bp)	Number of deduced amino acid residues	Molecular mass (kDa)	pI
HM131834	*lmGSTd1*	866	657	218	24.8	5.57
HM131840	*lmGSTs5*	680	609	202	23.1	5.72
HM131843	*lmGSTt1*	1100	696	231	26.6	7.63
HM131835	*lmGSTu1*	799	645	214	24.3	7.76

**Figure 1:**
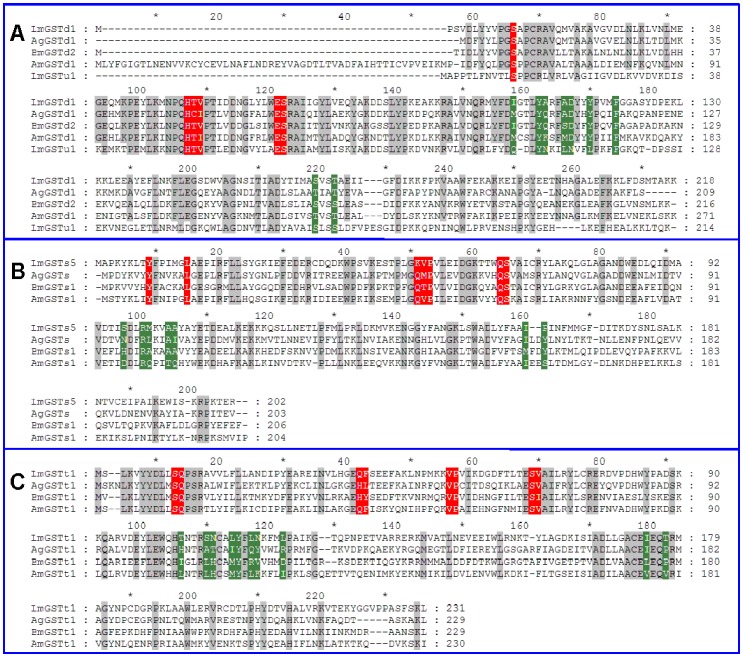
Similarity comparisons of the amino acid sequences of *L. migratoria* GSTs with GSTs from *Anopheles gambaie* (Ag), *Bombyx mori* (Bm), and *Apis mellifera* (Am). (A) Similarity comparisons of delta GSTs, including LmGSTd1 (ADR30117), AgGSTd1 (XP_313050), BmGSTd2 (NP_001036974), AmGSTd1 (NP_001171499.1), and LmGSTu1 (AEB91972.1). (B) Similarity comparisons of sigma GSTs, including LmGSTs5 (AEB91977), AgGSTs (P46428), BmGSTs1 (NP_001037077), and AmGSTs1 (NP_001153742). (C) Similarity comparisons of theta GSTs, including LmGSTt1 (AEB91980.1), AgGSTt1 (XP_311299), BmGSTt1 (NP_001108463), and AmGSTt1 (XP_624692). The conserved G-site residues are shaded in red, and the substrate binding pockets (H-site) are shaded in green.

*L. migratoria* GSTs show the characteristics of other insect GSTs. Specifically, multiple alignments of locust GSTs along with *A. gambiae*, *Bombyx mori*, and *Apis mellifera* GSTs revealed several key residues that are conserved across different insect orders (Fig. 1 A–C). The LmGSTu1 showed similar GSH binding site and the electrophilic-binding site to delta class GSTs (Fig. 1A). These residues constituted the putative GSH binding site and the electrophilic-binding site in the deduced amino acid sequence for the GSTs within their corresponding classes.

### Heterologous Expression and Purification of *L. migratoria* GSTs

**Figure 2:**
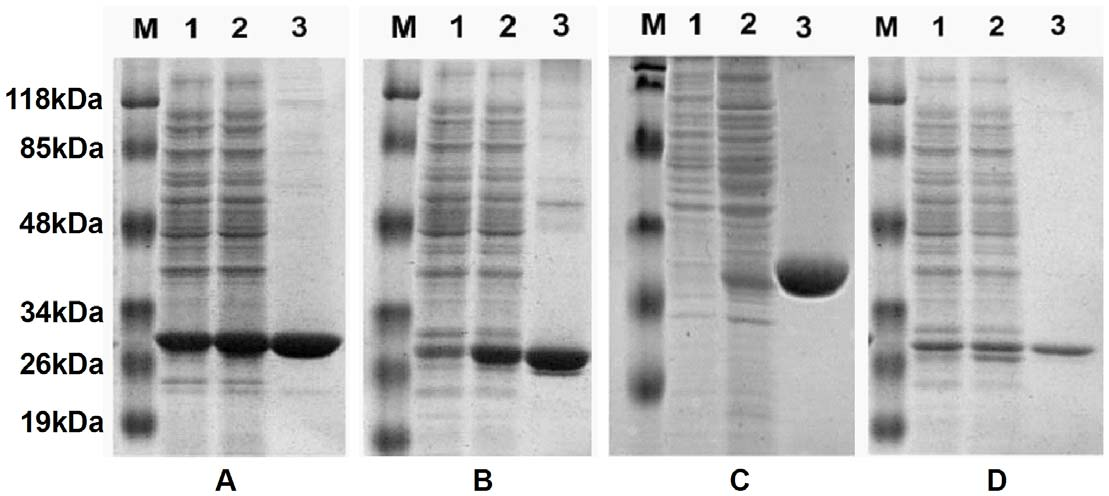
Analysis of the heterologously expressed and purified recombinant *L. migratoria* GST proteins by SDS-PAGE. (A) LmGSTd1, (B) LmGSTs5, (C) LmGSTt1, (D) LmGSTu1. The gel (12%) was stained with Coomassie Blue G-250. Lane M, protein molecular size marker. Lane 1, extract of BL21/JM109 carrying the expression vector for GSTs without IPTG. Lane 2, extract of BL21/JM109 carrying the expression vector for GSTs with IPTG induction. Lane 3, purified locust GSTs.

LmGSTd1 and LmGSTs5 were heterologously expressed in transformed *E. coli* JM109 with the pET-28a vector after 1 mM IPTG induction, whereas LmGSTt1 and LmGSTu1 were expressed in transformed *E. coli* BL21 (DE3). SDS-PAGE analysis of the cell lysate revealed that four LmGSTs were expressed in soluble forms (Fig. 2A–D). We finally obtained about 40–50 mg for each highly purified LmGSTs from *E. coli* cells with an approximate 1.3–5.6-fold purification. The recoveries were more than 50% (Table S2). The specific activities of the final preparations ranged from 1.77 to 14.29 µmol/min/mg protein. The molecular mass of purified LmGSTs were estimated to be approx. 27–30 kDa by SDS-PAGE (Fig. 2A–D). These are slightly larger than that predicted (23.1–26.6 kDa on the basis of its amino-acid composition) due to a 3.5 kDa vector-derived tag that is present on the N-terminal of the expressed proteins.

### Characterization of Recombinant *L. migratoria* GSTs

Kinetic analysis was carried out with 5 mM GSH and 0.15–0.5 mM different substrates at pH 7.5 and the results are summarized in Table 2. LmGSTd1 conjugated CDNB about 20- and 28,000-fold faster than LmGSTu1 and LmGSTt1, respectively. On the other hand, LmGSTs5 conjugated pNBC about 3-, 23-, and 109-fold faster than LmGSTd1, LmGSTu1, and LmGSTt1, respectively. Moreover, LmGSTs5 conjugated DCNB about 7.5- and 18-fold faster than LmGSTu1 and LmGSTd1, respectively (Table 2). The activities of four LmGSTs were undetectable when ECA was used as a substrate.

**Table 2:** Kinetic parameters of four *L. migratoria* GSTs heterologously expressed in *E. coli* as determined using selected substrates.

LmGST	CDNB		pNBC		DCNB	
	K_m_ (mM)	Vmax (nmol/min/mg)	K_m_ (mM)	Vmax (nmol/min/mg)	K_m_ (mM)	Vmax (nmol/min/mg)
LmGSTd1	0.5±0.025	5000±151	6.38±0.57	49.02±3.66	1.00±0.087	0.40±0.053
LmGSTs5	1.17±0.29	3889±962	0.47±0.083	156.19±18.44	1.73±0.24	7.41±0.96
LmGSTt1	4.73±0.35	0.18±0.03	7.85±1.08	1.43±0.13	ND	ND
LmGSTu1	0.25±0.025	250±21	1.58±0.17	6.77±0.74	0.73±0.064	0.98±0.10

Values are expressed as means ± SE of three independent experiments.

Km: concentration of substrate that produces half-maximal velocity.

ND: activity was not detected.

**Figure 3:**
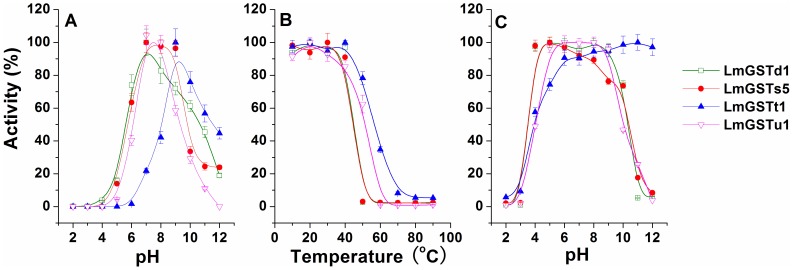
Enzymatic properties assayed with CDNB and GSH as substrates. The maximum value obtained was set to 100%. (A) Optimal pH of *L. migratoria* GSTs assayed using citrate–phosphate–borate buffer at various pH conditions. (B) Thermostability of *L. migratoria* GSTs. Thermostability determined by preincubation of the enzyme solution at various temperatures for 30 min before residual activity was assayed. (C) pH stability of *L. migratoria* GSTs. pH stability assessed by preincubation of the enzyme solution at various pH conditions at 4°C for 24 h before residual activity was assayed. Data are means and standard errors (SE) of three independent experiments (*n* = 3).

The enzymatic properties of LmGSTs were determined using the purified LmGST enzyme with CDNB and GSH as substrates. The optimal pH of the four GSTs ranged between pH 7.0 and 9.0 (Fig. 3A). Their thermostabilities were determined by preincubating each enzyme solution at various temperatures for 30 min before the residual activity was assayed. These GSTs were relatively stable during incubations at temperatures below 40°C (Fig. 3B). Theta GST is the most heat-tolerant protein; the residual activity was about 80% after the enzyme was incubated at 50°C for 30 min. The pH stability assessed by preincubation of the enzyme solution at various pHs at 4°C for 24 h before residual activity was assayed. The stabilities of these GSTs at different pH conditions varied significantly (Fig. 3C). Most LmGSTs retained activities at pH between 4.0 and 9.0, whereas LmGSTt1 retained more than 80% of its original activity at alkaline conditions.

**Figure 4:**
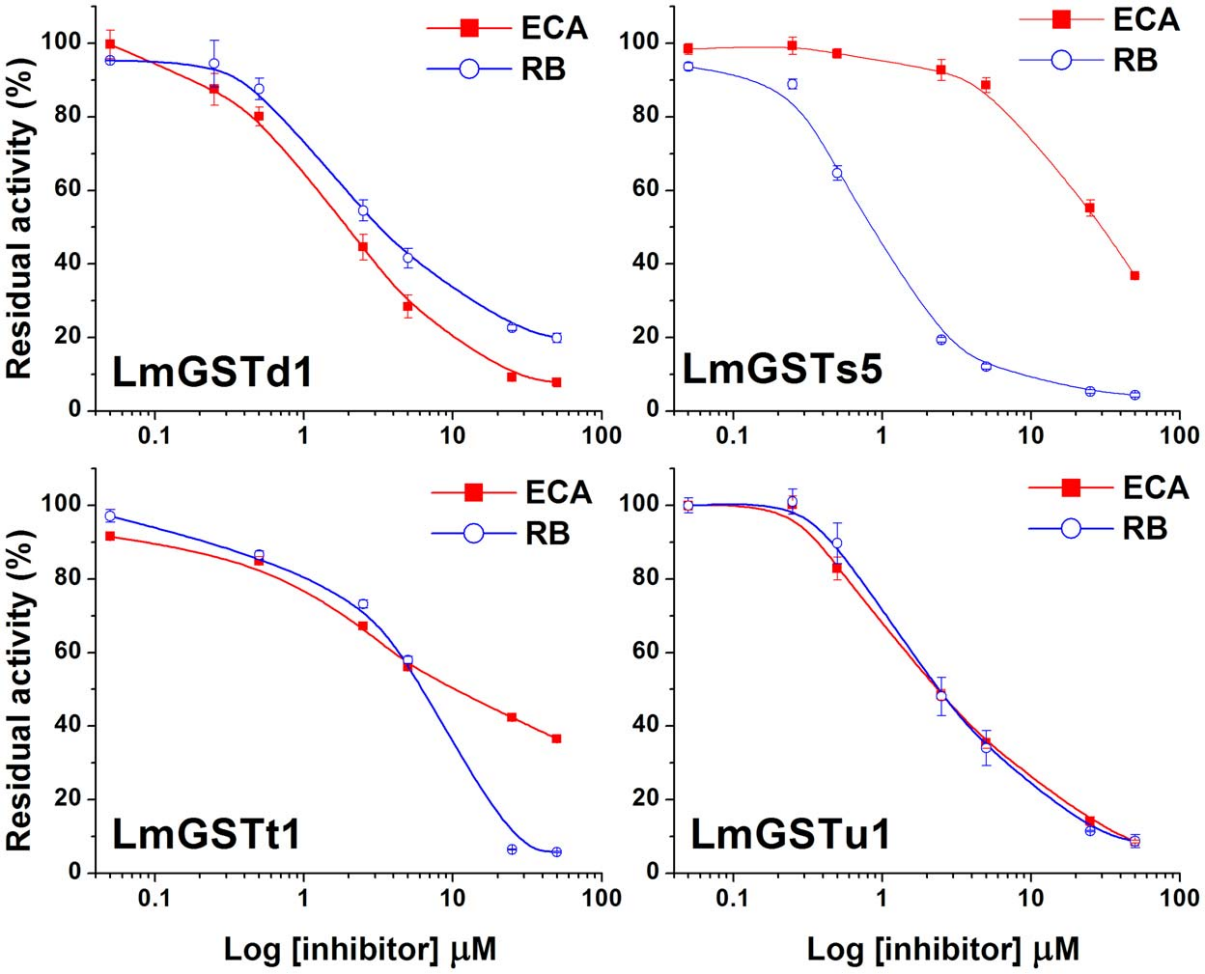
Inhibition of *L. migratoria* GSTs with ethacrynic acid (ECA) and reactive blue (RB). Data are means and standard errors (SE) of three independent experiments (*n* = 3).

The inhibitory effects of GST inhibitors including ECA and reactive blue (RB) on LmGSTs were examined with CDNB and reduced GSH as substrates. The results from inhibition experiments on LmGSTs indicated that both of the GST inhibitors inhibited the enzymes considerably, and that residual activity decreases with increasing concentrations of inhibitors (Fig. 4). ECA showed I_50_ values in the similar µM range against LmGSTd1, LmGSTt1, and LmGSTu1 (Table S3), whereas it showed relatively poor inhibition to LmGSTs5. In contrast, RB showed more than 34-fold greater potency against LmGSTs5 (Table S3). On the other hand, LmGSTt1 was relatively poorly inhibited by RB as compared with other *L. migratoria* GSTs (Table S3).

**Figure 5:**
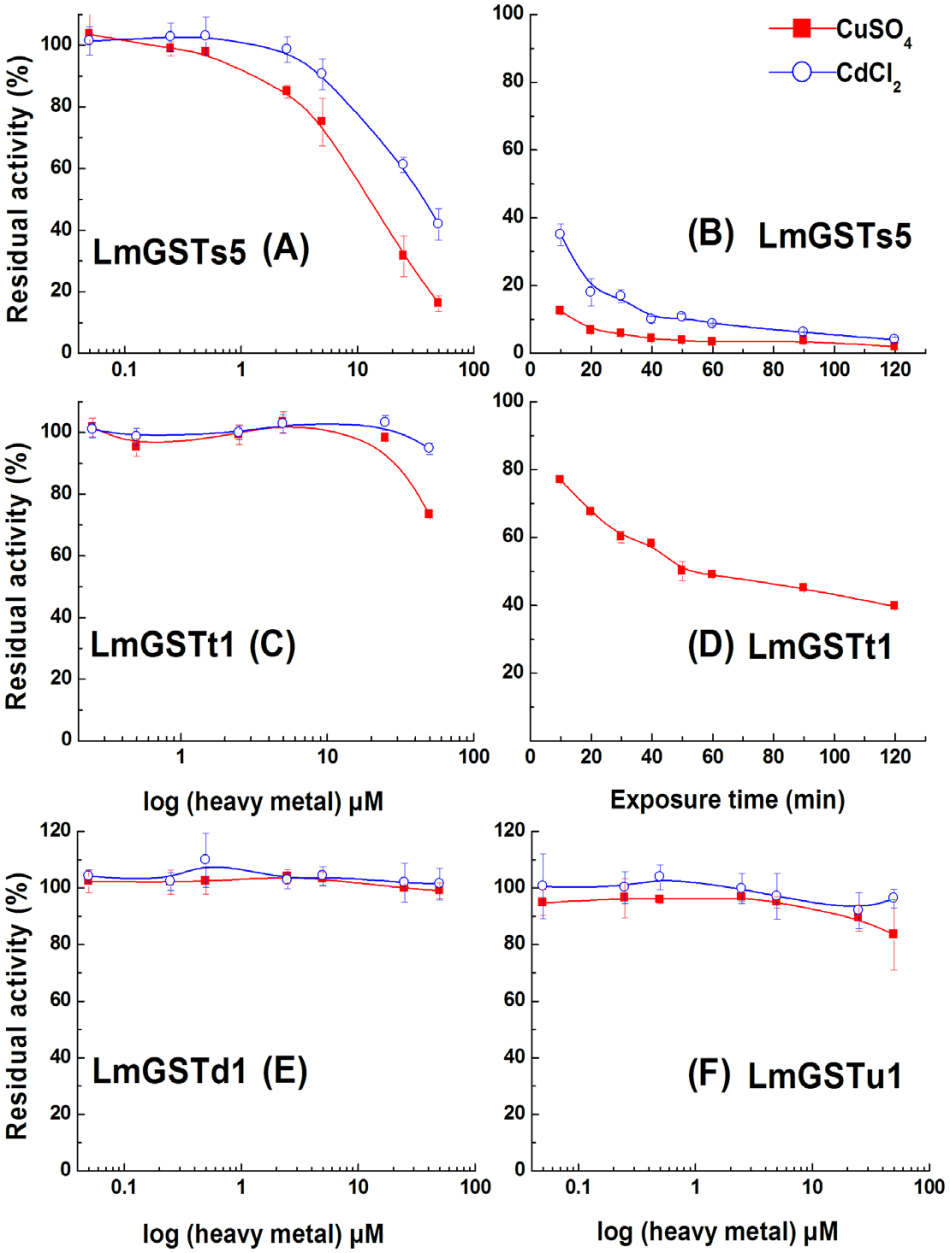
Effects of CuSO_4_ or CdCl_2_ on the activity of *L. migratoria* GSTs. (A, C, E, F) Enzymatic activity was measured in the presence of various concentrations of CuSO_4_ or CdCl_2_. (B, D) Enzymatic activity was measured by different incubation time in the presence of 50 µM CuSO_4_ or CdCl_2_. Data are means and standard errors (SE) of three independent experiments (*n* = 3).

Inhibition study showed that the recombinant LmGSTs5 was sensitive to heavy metals (Fig. 5). The residual activity of LmGSTt1 was decreased significantly in the presence of 50 µM CuSO_4_ (Fig. 5). The residual activities of LmGSTs5 and LmGSTt1 decreased significantly with exposure time in the presence of 50 µM heavy metals. While no significant inhibited effects were observed in LmGSTd1 and LmGSTu1 after heavy metals treatment (Fig. 5).

### Tissue-specific Expression Patterns of GSTs in *L. migratoria*

**Figure 6:**
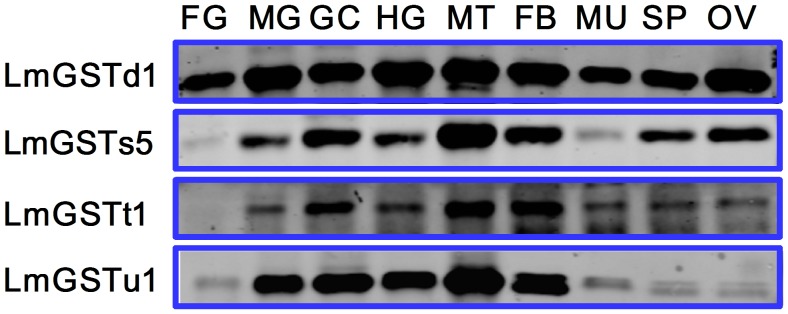
Tissue-specific expression patterns of the four GST proteins in *L. migratoria* as evaluated using western blot in foregut (FG), midgut (MG), gastric caecum (GC), hindgut (HG), Malpighian tubules (MT), fat bodies (FB), muscles (MU), spermary (SP), and ovary (OV).

Tissue-specific expression patterns of the four *L. migratoria* GST proteins were analyzed in nine different tissues, including foregut, midgut, gastric caecum, hindgut, Malpighian tubules, fat bodies, muscles, spermaries and ovaries by using western blot. Our results indicated that all the four classes of GST were expressed in all tissues examined, although there were some noticeable variations in expression levels among different tissues (Fig. 6). LmGSTd1 appeared to be strongly expressed in all these tissues. The maximum expression of four *L. migratoria* GST proteins was observed in Malpighian tubules and fat bodies. High expressions of GSTs were also detected in the midgut, gastric caecum, and hindgut. However, lower expression of LmGSTs5 was observed in the foregut and muscles. In contrast, LmGSTu1 was expressed mainly in the midgut, gastric caecum, hindgut, Malpighian tubules, and fat bodies, whereas the expression of LmGSTt1 was virtually undetectable in the foregut.

### Effect of *L. migratoria* GST Gene Silencing on Locust Susceptibility to Insecticides

**Figure 7:**
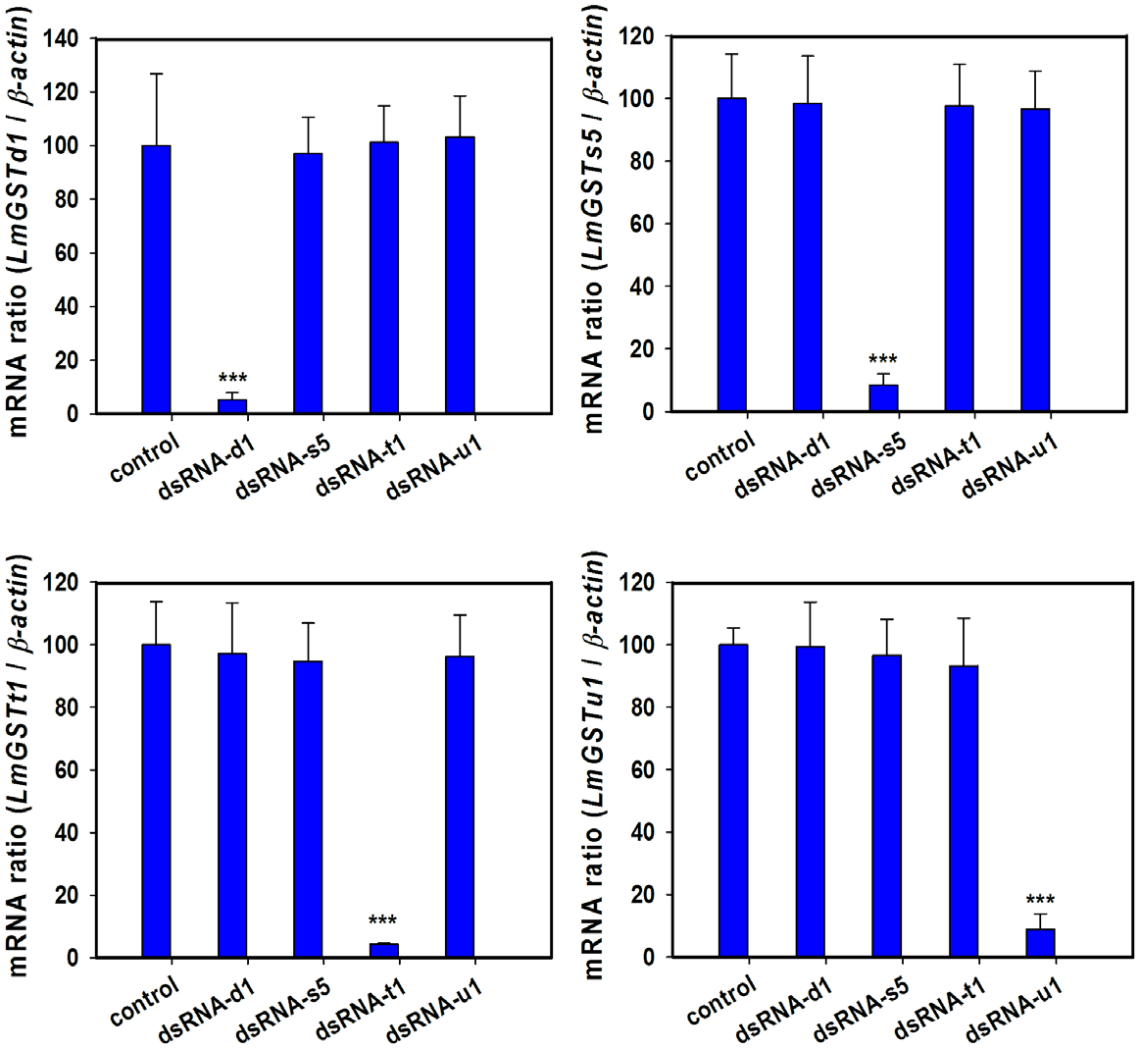
RNA interference efficiency of *L. migratoria* GSTs. RNAi analyzed by qRT-PCR at 24 h after injection of 3 µg double stranded RNA specific to each *LmGST* gene. The control locusts were injected with the same volumes of dsRNA of *GFP* gene. The mRNA levels in the control and treated groups were normalized using *β-actin* as a reference gene. Vertical bars indicated standard errors of the mean (*n*  = 3). Data are means and standard errors (SE) of three independent experiments (*n* = 3). Significant differences in the treated groups from their corresponding controls were assessed by *t*-test at * *P*<0.05, ** *P*<0.01, *** *P*<0.001.

Our qRT-PCR analysis of each *LmGST* transcript at 24 h after the injection of *L. migratoria* GST dsRNA showed a significant decrease as compared with that of each corresponding control, indicating an effective silencing of *L. migratoria* GSTs by RNAi. Furthermore, the injection of each of four dsRNA did not show any effect on the transcript level of other locust GST genes, indicating a specific silencing of each of these genes by RNAi (Fig. 7).

**Figure 8:**
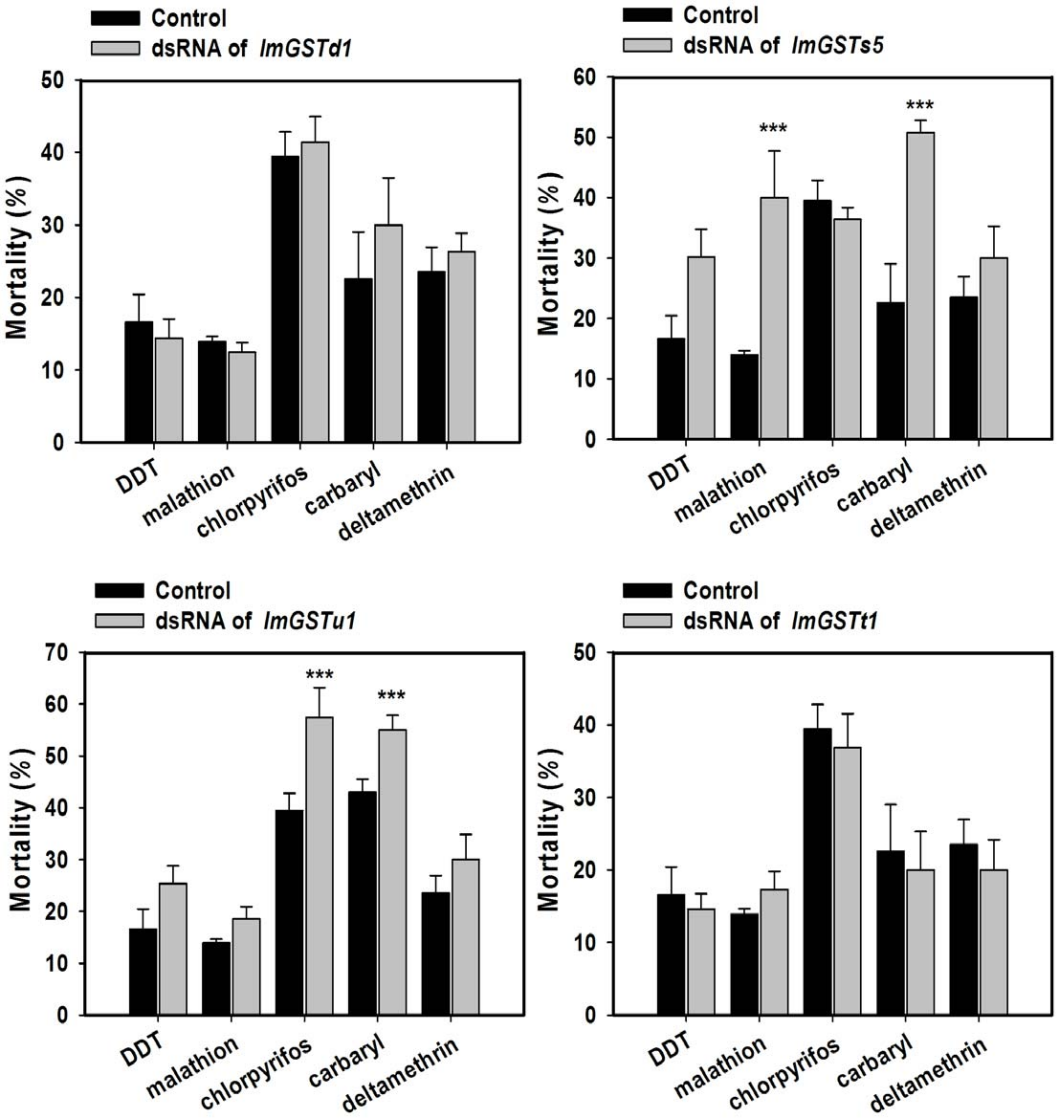
RNA interference effects of *L. migratoria* GSTs on the susceptibility of locusts to insecticides. Changes in the susceptibility of the locusts to different insecticides after the injection of locust GST dsRNA in second-instar nymphs. Insecticides bioassays were conducted 24 h after the injections by topical application. The mortalities of the locusts were assessed 24 h after the insecticides treatments. Data are means and standard errors (SE) of three independent experiments (*n* = 3). Significant differences in the treated groups from their corresponding controls were assessed by *t*-test at * *P*<0.05, ** *P*<0.01, *** *P*<0.001.

Insecticide bioassays showed that nymph mortalities in response to carbaryl treatment increased significantly (28 and 12%) after *LmGSTs5* and *LmGSTu1* were silenced, respectively. The nymph mortalities in response to malathion treatment increased from 13.9 to 40% after *LmGSTs5* was silenced, whereas the nymph mortalities in response to chlorpyrifos treatment increased from 39.4 to 57.5% after *LmGSTu1* was silenced (Fig. 8). Neither deltamethrin nor DDT showed significant changes in nymph mortalities after each of the four *LmGST* genes were silenced (Fig. 8).

## Discussion

It is well known that GSTs are a large family of multifunctional enzymes involved in the detoxification of hydrophobic and electrophilic toxicants including many drugs, herbicides and insecticides. There are at least 41 DmGSTs found in *Drosophila melanogaster* [20], 37 AgGSTs in *A. gambiae* [21], 12 AmGSTs in *A. mellifera* [22] and 23 BmGSTs in *B. mori* [23]. In our previous study, 10 GSTs were identified from *L. migratoria*. Among the 10 GSTs, nine were classified to three different cytosolic classes, including 1 in delta, 7 in sigma, 1 in theta, and the remaining one was designated as unclassified [17]. In this study, we selected one *LmGST* gene from each of the three classes and the unclassified group as the class representatives to biochemically characterize their heterologously expressed recombinant GSTs and functionally analyze their corresponding genes by using RNAi.

The cytosolic GSTs in most organisms are all dimeric with subunit molecular masses from 21 to 29 kDa [5]. Our predicted molecular masses of *L. migratoria* GSTs were in accordance with those previously reported. The kinetic parameters of *L. migratoria* GSTs were similar to corresponding GST classes from *Anopheles dirus* [24], *A. gambiae* [25], *Bemisia tabaci* [26], *B mori* [27], *Culex pipiens* [28], *D. melanogaster* [29], and *Hyphantria cunea* [30]. In the present study, we revealed optimal pH of the locust GSTs ranging between 7.0 and 9.0. Although most eukaryotic GSTs are known to have optimal pH ranging between 6.0 and 6.5 [31], some reports suggest that insect GSTs also have considerable activities at higher pH. For example, *Corcyra cephalonica* GST has an optimal pH 8.3 [4]. A recombinant GSTt1 expressed in *E.coli* from *B. mori* showed broadly optimal pH ranging between 4.0 and 9.0 [27]. Since it has been reported that locusts have alkaline internal environment after feeding [32], the GSTs with optimal pH in the alkaline range may be related to their functions.

The potency of ECA as GST inhibitor towards CDNB has been observed in earlier experiments with insects at similar inhibition level, e.g. *N. lugens* (40 nM), *Blattella germanica* (350 nM), *C. pipiens* (2.5 µM), *Spodoptera frugiperda* (150 nM), and *B. tabaci* (5.8 µM) [26], [28], [33]–[35]. ECA has a ketone moiety that forms a conjugate with GSH through a GST-catalyzed Michael addition reaction. This reaction is thermodynamically more favorable than the conjugation of CDNB to GSH via an addition-substitution reaction [36]. Thus, ECA can function to deplete GSH. Although the amino acid sequence comparisons indicated that both LmGSTs5 and LmGSTs3 are sigma-class GSTs, these GSTs showed unique inhibition profiles [18], suggesting that their substrate preferences may also be unique.

Copper (Cu^2+^) easily catalyzes the oxidation of the sulfhydryl group of GST (Christie and Costa, 1984). Cadmium (Cd^2+^) forms more stable coordination complexes with GST [37]. The inhibitory effect of Cu^2+^ and Cd^2+^ of the soluble GST forms has been reported previously [38], [39]. The addition of CuCl_2_ and CdCl_2_ 0.2 mM to the incubation mixture inhibits GST activity by 82 and 37%, respectively *in vitro* from rat liver [40]. GSTs in *Calystegia sepium* are inhibited by cadium ions only at concentrations higher than 100 µM [39]. However, little is known about which GST class is susceptible to inhibition by Cu^2+^ and Cd^2+^. Previous study presents the inhibition of a mu-class GST of the marine shrimp *Litopenaeus vannamei* by Cu^2+^ and Cd^2+^ [41]. Nevertheless, our study showed that sigma GST appeared to be the most sensitive to inhibition by both Cu^2+^ and Cd^2+^ in *L. migratoria*.

It is recognized that the expression of GSTs can change in different developmental stages and tissue types, and can be affected by feeding behavior and genetic factors of an organism [42]. Western blot analysis with antibodies generated against a *C. cephalonica* GST (CcGST) showed maximum expression of CcGST protein in fat bodies [4]. Our data are consistent with their findings by showing that the maximum expressions of the four *L. migratoria* GST proteins were observed in Malpighian tubules and fat bodies. The protein expressions of LmGSTd1, LmGSTs5, and LmGSTt1 in all tissues examined were consistent with their mRNA levels reported in our previous study [17]. However, high protein expressions of LmGSTu1 were observed in the hindgut and fat bodies other than midgut, gastric caecum, and Malpighian tubules. The mRNA of *LmGSTu1* was highly expressed in the latter [17]. Insects have long been known to excrete toxins via the Malpighian (renal) tubules, and the expressions of several GST genes have been found to be enriched in Malpighian tubules [43]. On the other hand, the fat bodies of insects are considered to be a major metabolic center and perform a large number of complex cellular functions [44]. High expression of LmGSTs in the midgut, gastric caecum and hindgut, which are generally exposed to a variety of xenobiotics through food, suggests that the LmGSTs might play an important role in detoxification of xenobiotics.

Indeed, the function of GSTs is generally considered to be the detoxification of both endogenous and xenobiotic compounds, and GSTs are involved in intracellular transport, biosynthesis of hormones and protection against oxidative stress [1]. Though GSH-dependent DDTase activity was discovered in several insect species, such as *Musca domestica* (housefly) [45], *D. melanogaster* [46], *A. gambiae* [9], *A. dirus* [47], and *Aedes aegypti* [48], detoxification of DTT was not observed by any of the four locust GST genes based on our RNAi experiments followed by DDT bioassay. Nevertheless, the detoxification roles of several *LmGST* genes against carbaryl, malathion, and chlorpyrifos have been evidenced by RNAi in this study.

Carbaryl is a member of the widely used carbamate insecticides. Like all carbamate insecticides, carbaryl acts as an inhibitor of acetylcholinesterase (AChE), an important enzyme involved in cholinergic neurotransmission in all animals including vertebrates and insects [49]. Carbaryl is not considered be metabolized via GST in previous study [50]. However, as validated by LmGSTs3 [18] and LmGSTs5 RNAi followed by insecticides bioassay, our results indicated that sigma GSTs in *L. migratoria* play a significant role in carbaryl detoxification. It has been well established that organophosphate (OP) insecticides are primarily metabolized by cytochrome P450 monooxygenases and hydrolases. However, there is a growing body of evidences that GSTs also play an important role in OP detoxification [51]. The action of GSTs on OP insecticides can lead to activation or detoxification [52]. Thiono-type OP insecticides, such as malathion, are not AChE inhibitors and require metabolic activation to become strong irreversible inhibitors of AChE *in vivo*. This activation mainly occurs through the action of cytochrome P450 enzymes, but subsequent reactions generally involve phase II reactions catalyzed by the enzymes such as GST. Our results indicate that LmGSTs5 is involved in malathion detoxification.

Chlorpyrifos, another widely used OP insecticide, is activated to chlorpyrifos oxon by cytochrome P450 enzymes and undergoes deethylation and dearylation, in human hepatocytes, or in *vivo* [53]. The metabolism yields a large number of metabolites which can be ultimately conjugated by GSH through GSTs. The locust mortalities after carbaryl and chlorpyrifos treatments increased after LmGSTu1 were silenced. It suggested that the unclassified LmGST played significant roles in both carbaryl and chlorpyrifos detoxification.

In conclusion, four GSTs representing different classes from *Locusta migratoria* were heterologously expressed in *E. coli* and biochemically characterized in this study. LmGSTt1 differed from other three GSTs at optimal pH, thermostability, and pH stability. The maximum expression of the four GSTs was observed in Malpighian tubules and fat bodies as evaluated by western blot. Our study suggest that sigma GSTs in *L. migratoria* play a significant role in carbaryl detoxification. LmGSTs5 also involved in malathion detoxification. The unclassified LmGST played significant roles in both carbaryl and chlorpyrifos detoxification. Studies such as this may lead to a more informed insecticide design strategy that takes into account the likelihood of degradation by the detoxification enzymes of the pest insect. However, genome-wide search of all the GST genes in *L. migratoria* followed by functional analysis is needed in future research.

## Supporting Information

Figure S1 The specificity of the primary antibody of four *L. migratoria* GST proteins was detected using western blot.
(TIF)

Table S1 Primers for PCR of GST genes contained restriction enzyme sites of *L. migratoria.*
(DOCX)

Table S2 Summary of the purification of LmGSTs from *E. coli* cells.
(DOCX)

Table S3 Median inhibition concentrations (I_50_) of ethacrynic acid and reactive blue against LmGSTs.
(DOCX)
