# Supplementary figures and images for: Characterization and Functional Analysis of Four Glutathione S-Transferases from the Migratory Locust, Locusta migratoria
Source: PLoS One. 2013 Mar 7;8(3):e58410. doi: 10.1371/journal.pone.0058410 (PMC3591310; doi:10.1371/journal.pone.0058410)

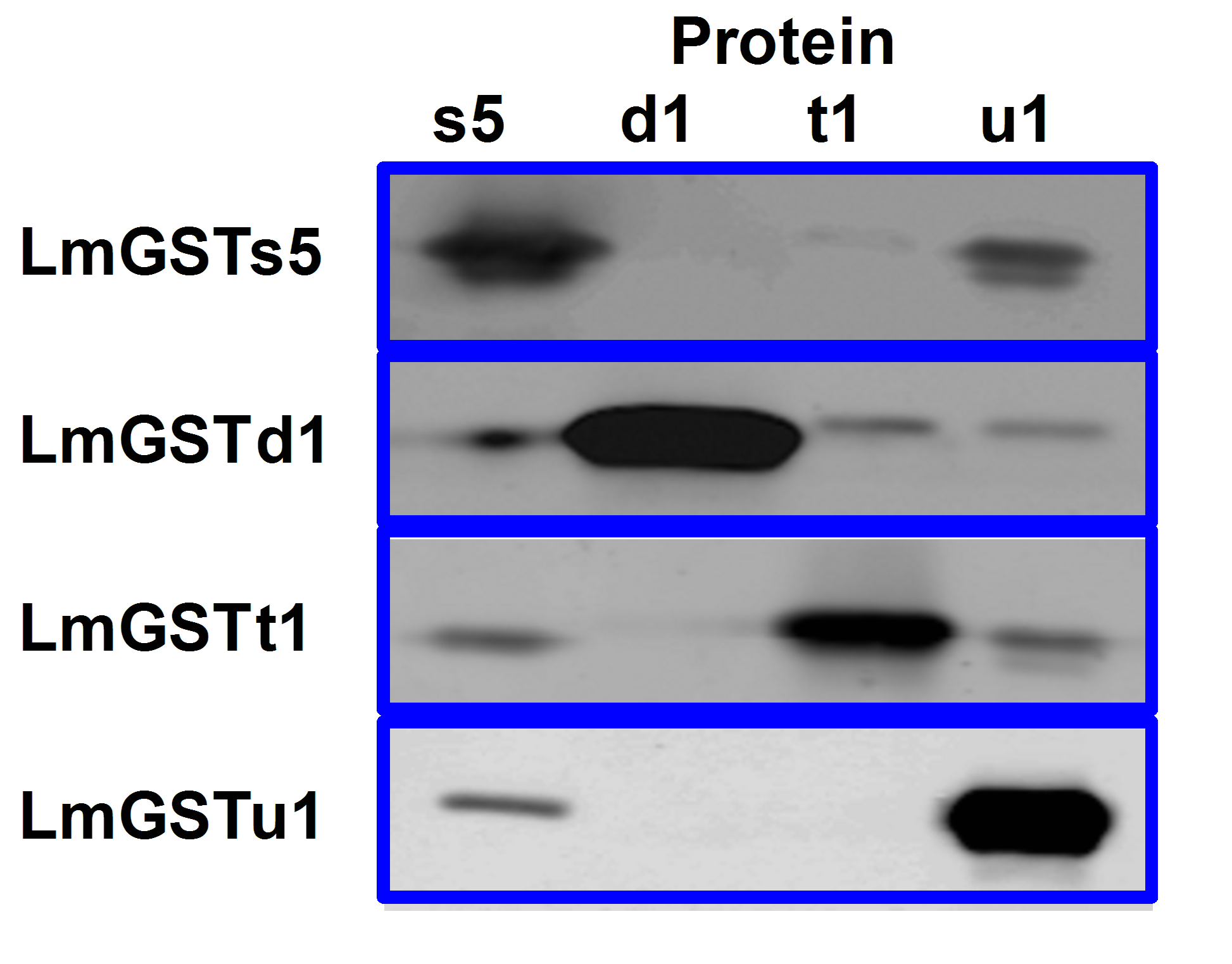

Supplement: Figure S1 — The specificity of the primary antibody of four L. migratoria GST proteins was detected using western blot. (TIF) [file pone.0058410.s001.tif]
