# Supplementary material for: Characterization and Functional Analysis of Four Glutathione S-Transferases from the Migratory Locust, Locusta migratoria
Source: PLoS One. 2013 Mar 7;8(3):e58410. doi: 10.1371/journal.pone.0058410 (PMC3591310; doi:10.1371/journal.pone.0058410)
Supplement: Table S1 — Primers for PCR of GST genes contained restriction enzyme sites of L. migratoria. (DOCX) [file pone.0058410.s002.docx]

Table S1 Primers for PCR of GST genes contained restriction enzyme sites of *L. migratoria*

| GenBank | Gene | Primers | Sequence(5'-3') | restriction enzyme |
| --- | --- | --- | --- | --- |
| HM131834 | *LmGSTd*1 | P-Forward | CTCGGATCCTTCATCACCATG | *Bam*H I |
|  |  | P-Reverse | GGCAAGCTTTCAGTCTTACTT | *Hind* III |
|  |  | ds- Forward | taatacgactcactatagggGCAAAGAAGAGAGCATTGGTGA |  |
|  |  | ds- Reverse | taatacgactcactatagggGCTCCTGCGTGATTAGTTTCTTC |  |
|  |  | Q- Forward | GTAGTTCTGTCGCCGGTTATACTG |  |
|  |  | Q- Reverse | AATACAAATGCAATCTCAAAATGGA |  |
| HM131840 | *LmGSTs*5 | P-Forward | TTAGGATCCATGGCACC | *Bam*H I |
|  |  | P-Reverse | CAGGAGCTCTCATCTCTC | *Sac* I |
|  |  | ds- Forward | taatacgactcactatagggACATGGCAGTTGACACAATATCAG |  |
|  |  | ds- Reverse | taatacgactcactatagggTGGTCTCTTGCTAATCCACTCCTT |  |
|  |  | Q- Forward | AGATGGCACCAAAATACA |  |
|  |  | Q- Reverse | CAAAGCCAGAGCACTAAT |  |
| HM131843 | *LmGSTt*1 | P-Forward | GTCGGATCCATGTCACTG | *Bam*H I |
|  |  | P-Reverse | GACGAGCTCACAATTTAC | *Sac* I |
|  |  | ds- Forward | taatacgactcactatagggTGGCAAATGACATCCCTTAT |  |
|  |  | ds- Reverse | taatacgactcactatagggTGGCAAATGACATCCCTTAT |  |
|  |  | Q- Forward | TGGTAAGACCTGGAAAAC |  |
|  |  | Q- Reverse | ATCTACCAACGTGTTTCC |  |
| HM131835 | *LmGSTu*1 | P-Forward | TGCGAGCTCACAGCAATGG | *Sac* I |
|  |  | P-Reverse | TGAAAGCTTCTACTTCTGCGT | *Hind* III |
|  |  | ds- Forward | taatacgactcactatagggCGCCTGGTCCGTTTAGTG |  |
|  |  | ds- Reverse | taatacgactcactatagggTCCAGCCCTTCGTTCACTT |  |
|  |  | Q- Forward | GACACGCCACATCTGAAAC |  |
|  |  | Q- Reverse | GCTGCTACTTCTGCGTCAA |  |
