# Supplementary material for: Characterization and Functional Analysis of Four Glutathione S-Transferases from the Migratory Locust, Locusta migratoria
Source: PLoS One. 2013 Mar 7;8(3):e58410. doi: 10.1371/journal.pone.0058410 (PMC3591310; doi:10.1371/journal.pone.0058410)
Supplement: Table S2 — Summary of the purification of LmGSTs from E. coli cells. (DOCX) [file pone.0058410.s003.docx]

Table S2 Summary of the purification of LmGSTs from *E. coli* cells

|  | Total protein (mg) | Total activity (µmol/min) | Specific activity (µmol/min/mg protein) | Purification Fold | Recovery (%) |
| --- | --- | --- | --- | --- | --- |
| LmGSTd1 | 42.06 | 96.09 | 2.28 | 1.29 | 51.19 |
| LmGSTs5 | 41.40 | 91.24 | 2.20 | 1.60 | 57.22 |
| LmGSTt1 | 39.96 | 570.95 | 14.29 | 5.60 | 74.60 |
| LmGSTu1 | 49.26 | 87.16 | 1.77 | 1.62 | 59.18 |
