# Supplementary material for: Characterization and Functional Analysis of Four Glutathione S-Transferases from the Migratory Locust, Locusta migratoria
Source: PLoS One. 2013 Mar 7;8(3):e58410. doi: 10.1371/journal.pone.0058410 (PMC3591310; doi:10.1371/journal.pone.0058410)
Supplement: Table S3 — Median inhibition concentrations (I50) of ethacrynic acid and reactive blue against LmGSTs. (DOCX) [file pone.0058410.s004.docx]

Table S3 Median inhibition concentrations (I_50_) of ethacrynic acid and reactive blue against LmGSTs

|  | I_50_ (μM) | |
| --- | --- | --- |
|  | RB | ECA |
| LmGSTd1 | 4.58 | 2.20 |
| LmGSTs5 | 0.98 | 33.77 |
| LmGSTt1 | 12.40 | 4.70 |
| LmGSTu1 | 3.40 | 3.27 |
